# Anthropometric Studies on the Turkish Population - A Historical Review

**DOI:** 10.4274/Jcrpe.957

**Published:** 2013-03-21

**Authors:** Olcay Neyzi, Hatice Nurçin Saka, Selim Kurtoğlu

**Affiliations:** 1 İstanbul University İstanbul Faculty of Medicine, Pediatric Endocrinology, İstanbul, Turkey; 2 Erciyes University Faculty of Medicine, Pediatric Endocrinology, Kayseri, Turkey

**Keywords:** Turkish population, anthropometry, growth, history

## Abstract

A historical review of anthropometric studies conducted on Turkish children and adults is presented. In view of observed differences in growth status between children of different societies, the need for local reference standards and the methodology to be used for such studies have been stressed. The importance of local studies in reflecting the state of health and nutrition both in children and adults has also been mentioned. While a number of studies in children cited in this paper are designed to compare the growth of children from different socioeconomic levels, other studies aim to establish local reference data for Turkish children. While the historical studies in adults aim to define racial characteristics, the more recent studies aim to bring out nutritional characteristics with emphasis on increasing frequency of obesity.

**Conflict of interest:**None declared.

## INTRODUCTION

The first documented growth studies in Europe date back to Buffon’s “Supplements to the Natural History” published in 1777. Montbeillard’s longitudinal measurements of his son from birth to age 19 years, the first growth study of its kind, are also included in this work. However, it was not until the end of the 19th century that the foundation for contemporary growth studies was established with the work of Franz Boas (1), who was the first to point out variations in tempo of growth. Boas, with his subsequent work and use of graphic methods has also inspired more recent studies, such as the Harvard Growth Study (2,3).

For adults, data on height in Western European and North American populations are abundant from 1700 on and show positive and negative trends over time until significant positive secular trends are noted in the first thirty years of the twentieth century in Western Europe and much earlier in the United States (4). While there are some ethnic groups in which mean adult heights differ significantly from the Western European and North American values, presumably due to genetic factors, systematic genetic influences appear to have little impact on mean heights for most population groups. Today, the mean heights of well-nourished Western Europeans, North American whites and North American blacks are nearly identical. The importance of genetic factors has undoubtedly been overemphasized by 19th century scholars and efforts to describe ethnic groups with reference to anthropometric characteristics, so popular in that era, are no longer considered to be as pertinent (5). It is only recently, through studies which account also for the strong impact of environment on growth and maturation, that clear genetically controlled differences between populations have been identified (6,7,8,9).

No anthropometric data on the Turkish population are encountered in the archives prior to the turn of the century. This paper aims to review the available documents on anthropometric measurements, excluding those pertaining to the newborn, on the Turkish population in chronological order, thus also bringing to light the changes in these values as well as in perspectives that have occurred over time. For purposes of clarity, anthropometric studies on children and those on adults will be treated under separate headings.

**Anthropometric Studies on Turkish Children**

The first published study on the anthropometric measurements of Turkish children was performed by Kansu in 1917 (10). The author measured heights and weights in a group of 281 school children (125 girls and 156 boys) in the city of Bursa. The children were between 7 and 20 years of age, and of middle-class families. In the introductory paragraph of his paper, the author states that the Ministry of Education of the Ottoman Empire attached importance to the assessment of growth and development and had distributed a “Medical Examination Form”, which also included body measurements, to all schools in 1915.

However, probably due to the political upheavals in the country which culminated in the founding of the new Turkish Republic, it was only a decade later that, in collaboration with the Ministry of Education, stature, weight and chest circumference measurements were performed on a group 4000 Turkish, 220 Greek, 1600 Armenian, 1340 Jewish and 720 “Levantine (refers to persons of European origin residing in Ottoman/Turkish domains)” children aged 9 to 18 years in Istanbul. It is unfortunate that in this study the results were analyzed for boys and girls together, without any sex differentiation (11).

Starting in 1938, studies based on groups of children whose characteristics were more precisely defined began to be published. Alantar (12), in the introductory remarks to his paper which was also presented as a report at the first National Congress of Pediatrics in Turkey, emphasized the importance in growth studied for adequate sample size, separate evaluation of two sexes, the need for exact calculation of chronological age for each child and the use of standard measurements methods. In Alantar’s study, height and sitting height were measured in 5412 girls and 4888 boys between 1 month and 12 years of age who attended the outpatient clinic of a large pediatric hospital in Istanbul. The results, expressed as mean values, were compared with data from other countries. Another study, in which weight, height, head and chest circumferences were measured in 6774 girls and 6462 boys from birth to 16 years who attended a clinic in a less developed district of Istanbul, was also presented in the same Congress of Pediatrics (13).

During the same years, several studies on school children were undertaken. These included, in addition to height and weight, anthropometric measurements such as limb length, span, sitting height and hand length, as well as assessment of racial characteristics such as cranial and facial measurements, eye shape and color, nose profile, hair and skin color (14,15,16,17,18), These studies revealed that the majority of Turkish children possess a face which is either narrow or of medium width, a long, narrow, straight nose and skin, hair and eyes of medium color. These children were also evaluated by anthropological indices. Kinay (18) reported that children living along the coast were taller than those from inland areas.

Ilbars (19) performed several anthropometric measurements on 200 children between 8 and 13 years in an effort to establish a relationship between body shape and athletic activities. The author reported that children of a macroskelic body type performed better in physical activities such as running and jumping, while the brachyskelics were stronger (better in pulling and similar activities). Thus, the investigator concluded that body shape should be taken into consideration in selection of athletic activities for the individual child.

The first growth study in which the results were expressed as means including standard errors for each chronological age belongs to Yalim (20), who reported height and weight measurements in 12277 (6144 boys, and 6133 girls) children between 7 and 18 years of age, attending schools in various districts of the city of İstanbul. The measurements were performed by the school doctors or teachers. The study showed that with the exception of age groups 11-13 years, the boys were slightly taller and heavier than the girls.

Mean values for heights and weights reported by the above authors at all ages are close to the 5th percentile lines of the international standard based on the United States National Child Health Study (NCHS) (21). The retardation is less marked for weight values.

Eckstein et al (22) assessed weight gain in 66 boys and 59 girls in the first year of life. At one year of age, weight was essentially comparable in all infants, regardless of birth weight. It was shown that weight gain in infancy is higher in infants born with relatively lower weights. Boy infants also showed a higher gain compared to girls.

Between 1954 and 1957, Bostanci carried out four studies in Ankara on 1679 school children (832 boys, 847 girls) between 9 and 16 years of age (23,24,25,26). The size of the sample for each sex in each chronological age group varied between 100 and 123. These studies were the first of their kind in Turkey in which the methodology is described in detail; a total of 35 anthropometric measurements were performed on each child and several indices were used in the evaluation. Height measurements showed that the girls were slightly taller than the boys at ages 11-14 years. Starting at age 15 years, the boys became significantly taller. Mean trunk length was also greater in the boys except in age groups 11 to 15. Brachyskely was a feature common to both sexes at all ages. However, the girls had higher trunk indices at all ages except at age 9 and the difference between the two sexes increased with age. These results showed that the girls were relatively more brachyskelic as compared to the males. Foot width and foot length were greater in boys at all ages. The study also showed that cessation of growth in foot width and length occurred at an earlier age as compared to the femur and the tibia. The foot also became thinner with age. Mean values for foot index were higher in boys in all age groups. Hand index was also higher in the boys except for age groups 9 and 10. In both sexes, the lower limb values (leg length, foot length, foot width) were consistently higher than the corresponding measurements of the upper limbs (upper and lower arm lengths, hand length, hand width). Attainment of adult values in limb lengths occurred in the hand, forearm and upper arm in the upper extremity; and in the foot, lower leg and upper leg in the lower extremity, in respective order. The author also reported values on shoulder width, hip width, chest width, chest depth, and chest circumference measurements.

Soysal et al (27) stressed the importance of nutrition and other environmental factors on growth and the need to select groups of children growing under optimal conditions in establishing the local growth standards of a community. These authors reported height and weight measurements in infants and children from birth to 8 years of age who were seen in private pediatric practice. The study was based on 4973 measurements in the girls and 6260 measurements in the boys. The results of this study showed that the mean values were almost identical to those of North American white children and higher than height and weight measurements reported for Turkish children in previous studies. Other workers in the 1960’s reported data showing differences in anthropometric measurements in school children of different socioeconomic background and stressed the importance of socioeconomic level and nutrition on growth performance (28,29,30,31).

Koksal et al (32) studied a group of children between 2 months and 5 years attending a factory day care center. In this mixed longitudinal/cross-sectional group, height measurements conformed to international standards until 6-9 months of age and showed a downward trend thereafter, falling between the 3rd and 10th percentiles at age 5 years. Weight measurements were closer to the 50th percentile during and after infancy.

Oral (33) reported measurements on children living in a rural area in the vicinity of Ankara and who were followed by a University team. The author reported retardation in growth in all parameters starting at age 3 months. However, after the first year of life, growth tempo in weight and head circumference followed a line parallel to standard curves, while height velocity remained subnormal for another year.

Neyzi et al (34,35) determined the anthropometric parameters in children aged 9 to 17 years of different socioeconomic groups and reported large differences.

In 1975, Onat (36) reported the results of a longitudinal study on height and weight performed on two groups of girls. One group consisted of girls attending a school in a semiurban district of İstanbul, while the girls in the second group were of higher socioeconomic level. Both groups, starting at ages 86/12 and 911/12 years, were followed for 7 years. A significant retardation in growth relative to children from higher socioeconomic levels was noted in the girls from a lower socioeconomic background. The author also compared height and weight values in the total group with those reported by Yalım in 1940 and found a height increase of 4.4 cm at age 8, indicating an important secular trend over a period of 30 years. While some increase was noted also in weight, the secular trend in weight was not significant. The girls in Onat’s study were also 2 cm taller than their mothers.

In 1979, Neyzi et al (37) reported data on growth in infants and children from 1 to 36 months of age and of low socioeconomic level, attending a maternal and child health center (MCH) in İstanbul. Despite regular attendance to the Center, increase in head circumference as well as in weight and height showed a lag at 6, 9, and 12 months of age, respectively.

Genetic variations relating to both size and tempo of growth are known to exist among different populations, indicating a need for local growth standards. On the other hand, it is also well known that poor nutrition and other unfavorable environmental factors have an important impact on growth. Since a significant proportion of children in the developing regions of the world cannot escape the effect of a suboptimal environment on growth, the anthropometric reference standards in such regions need to be derived from selected samples of the population who provide their children with the requirements for optimal growth. With the above considerations and aiming to establish local norms for Turkish children, Neyzi et al (38,39) reported heights and weights in 3606 healthy Istanbul children of ages between 1 month and 18 years (1755 girls and 1851 boys) of high socioeconomic level born between the years 1955 and 1965. For infants, another criterion for inclusion in the series was periodic visits to a pediatrician. Longitudinal data for children up to 8 years were collected retrospectively from the files of two pediatricians. Data on children from 9 years on were based on measurements obtained in schools and were cross-sectional. Exact chronological age was known in all these subjects and all measurements were performed using standard equipment and methodology. Since the means and standard deviation (SD) values were derived from measurements taken at exact ages or within negligible distances from exact chronological ages, correction of the SD was not attempted. The percentile curves fitting the points obtained as described above were assessed in three different ways: a) hand fitting; b) 3rd and 4th degree polynomial regression analysis applied to all age groups; and c) regression analysis after classifying the subjects into three age categories (0-3 years; 4-10 years; 11-18 years). A good fit could be obtained by applying a second degree polynomial curve to the 0-3 year’s age group, a first degree (a line) to the 4-10 years age group, and an exponential curve to the 11-18 years age group. The growth charts prepared by Neyzi et al (38) have been in use in the assessment of Turkish children living in Turkey or in Europe for nearly three decades.

In a study (40), children of Turkish emigrant workers in Sweden who were born and reared in Sweden were compared with those who were born in Turkey and emigrated in childhood, as well as with Swedish children and with Turkish children of well-off families in Turkey and it was found that Turkish children who were born and reared in Sweden were shorter than Swedish children, but also shorter than the children of well-off families in Turkey. Those who had emigrated to Sweden in childhood were short on arrival but showed significant catch up. This study demonstrates the importance of environmental effects on growth.

Several other studies on anthropometric measurements, including foot measurements, head length/stature ratios, pelvic width and determination of height velocity on Turkish children of different socioeconomic level and living in different parts of the country have been published in the years prior to 2000 ?(41,42,43,44,45,46,47,48,49,50,51,52,53,54,55,56,57,58,59,60). Most of these studies were performed by pediatricians and aimed to show differences in body size in children of different socioeconomic levels or in those living in urban as opposed to rural settings.

Studies on secular trend in Turkish children are limited. Neyzi et al (61), by reviewing available data on Turkish children in the past 50 years, reported significant differences in height for age in Istanbul city children. As also observed in other countries, secular trend is mainly due to improved growth performance of children from lower socioeconomic classes (62,63,64). Simsek et al. (65) and also Ozer (66,67), in their studies on school children in Ankara, also reported significant increments in height during the past decades. Ozer’s results demonstrate increments in height, leg length, and weight, which are more prominent in boys. No significant change in sitting height was found.

Among the several studies on heights and weights conducted on the Turkish pediatric population since 2000, studies by two groups of researchers, those working in Istanbul and in Kayseri, deserve mention, since both groups worked on study groups of adequate size and composition to constitute a reference sample, and both groups applied standard and up-to-date methodology for measurements and analysis of the results.

The İstanbul study was based on 11 664 height and 11 635 weight measurements on 2129 (1100 boys and 1020 girls) school children (of ages 6 to18 years) (68) and also included height, weight and head circumference measurements on 2391 boys and 2102 girls followed at the Well Baby & Child Clinic of the Pediatric Department from birth to age 5 years (69). The results of the two age groups were combined to derive the updated reference percentile growth charts for weight, height and head circumference and for body mass index (BMI) in Turkish children (70). The height results compare well with the North American white children included in the NHANES III study (71). Weight measurements and BMI results indicate that Turkish children are prone to be become overweight from an early age on and that obesity reaches significant proportions starting in prepubertal years. Another noteworthy finding was the relatively increased head circumference values as compared to western standards, which were thought to reflect a genetic characteristic, since the study group included only healthy and well-nourished subjects.

The studies on Kayseri children were conducted on healthy children and consisted of 2785 boys and 2942 girls aged 6 to 18 years and of 1472 boys and 1491 girls aged 0 to 84 months (72,73). Height and weight measurements were taken, and percentile growth charts to serve as references were constructed. The results, compared with those obtained for İstanbul children, revealed that in the first year of life, Kayseri boys and girls had lower weight and height values, but that they became slightly taller and heavier than İstanbul children between ages 4-12 years. In older age groups, they were slightly shorter and of lower weight than the Istanbul children. Some members of the Kayseri group, Malkoc et al (74), studied the effect of altitude on height, weight and BMI of children aged 6-14 years and reported that children residing at higher altitudes were shorter and leaner than their Istanbul or Kayseri counterparts. However, the authors considered socioeconomic level also as a possible factor influencing the results.

The Turkish Demographic and Health Survey (TDHS) data also provide anthropometric information on Turkish infants and children under age 5 years (75). Since 1968, TDHS has been conducted every 5 years and provides data on samples representative of the whole of the Turkish population. These data show very significant improvements in social as well as in health indicators over time. The latest TDHS report, the proportion of stunting (height <-2SD) is given as 13% and that of underweight infants/children as 1.7% (weight <-2SD). Frequency of low weight-for-height ratio is given as 0.8%. Compared to previous years, a significant decrease is noted in frequency of low weight-for-age and in low weight-for-height figures in young Turkish children, revealing a potential increase in obesity. Proportion of low birth weight infants (birth weight <2500 g) is given as 11%. A recent Ministry of Health nationwide survey conducted on 11 387 school children aged ?6-10 years revealed that the frequency of underweight children (Z score <-2SD) was 2.4%, while that of overweight (Z score >+2SD) was 4.9%. Frequency of stunting (Z score <-2SD) was reported as 5.0%. Figures for BMI, waist circumference (WC) and WC/Ht ratio are also given in the report.

Most other recent reports on measurements are local studies with limited numbers of subjects (76,77,78,79,80,81,82,83,84,85,85,87,88,89,90,91). Of these, two studies (86,88), by measuring height and weight in children working as industrial workers, document the negative effect of child labor on growth. Reports on head circumference have also been published in recent years (89,90). Of these, Elmali et al (90) have produced percentile charts for head circumference in Kayseri children aged 0 to 84 months. In another study, heights and weights of primary school children from three different regions in Turkey were measured and the results revealed significant differences which were attributed to differences in socioeconomic conditions (91).

In the past decade, several groups of investigators have reported BMI and body composition values in Turkish children from various regions of the country (70,92,93,94,95,96,97,98,99,100,101). Of these, the studies on İstanbul children and those from Kayseri (70,74,92,100) are based on samples of adequate size, and the BMI percentile charts produced in these studies can be advocated to be representative of the population. It should be noted that as compared to NHANES III data (71), obesity stands out as an important problem in Istanbul children, starting in prepubertal years and reaching high proportions after puberty (overweight: 25% in boys and 15% in girls; obese: 4.0% in males and 1% in girls) (70,92). The study on Kayseri children however revealed different results. While BMI values in Kayseri children were higher compared to WHO standards in the first 5 years of life and frequencies for overweight and obesity were reported as 10% and 4.9% respectively (97,100) in this age group, BMI values in older children were shown to be relatively low as compared to European, North American and Istanbul children. These results indicate that in Turkey, children of high socioeconomic groups and possibly those growing up in a big city are more prone to become overweight/obese in adult life.

There are also studies on measurements of body segments and relationships between arm span and height in Turkish children (102,103,104,105). Studies aiming to establish reference values for WC and also for neck circumference, measurements which would serve as indicators for central obesity, have also been realized, and percentile charts for these values in boys and girls have been constructed (106,107,108,109) for these measurements. Several studies on skinfold thickness, upper midarm circumference (UMAC) and the relationship of these values to undernutrition ?and overnutrition have also been realized by pediatricians in Turkey (110,111,112,113,114,115,116,117). Measurements of interpopliteal distance and reference values for this indicator, which would serve in the diagnosis of rickets syndromes in young children, have also been reported (118).

Studies on skeletal maturation and growth at puberty in Turkish children also need to be included in this review. Findings related to age and progression of sexual development in Turkish girls and boys have been reported by Neyzi et al (119,120). Neyzi et al (121) have also analyzed the relationships between age at menarche and height, body weight, weight/height ratio, skinfold thickness and bone age and have reported significant relationships between age at menarche and body weight and also between age at menarche and skeletal age. In a subsequent study, it was shown that while BMI continued to be a significant factor influencing age of menarche, differences in menarche age among socioeconomic classes disappeared with improvement in socioeconomic conditions over time (122). Kinik et al (50) have reported a significant relationship between height and skeletal age in boys of pubertal ages. Onat and Ertem (123) reported no significant relationship between menarche age and final height or weight in the 114 girls they have followed for 7-9 years, but when height at menarche was expressed as a percentage of final height, it was found that this value was significantly lower in girls who had menarche at a younger age. Onat and Iseri (124) also studied relationships between skeletal maturation in the digital bones of the hand and development of secondary sex characteristics and reported a lower growth potential in girls who had more advanced bone maturation. This same author, using his longitudinal data on girls, also evaluated the efficacy of different methods used in estimation of final height and reported that both Bayley-Pinneau and Tanner-Whitehouse methods were reliable (125,126,127). Bundak et al (128), who investigated the relationships between onset of puberty, progression of puberty and final height in 1112 boys between ages 8-18 yrs, reported age of onset of puberty as 11.6±1.2 yrs, height at onset as 146.1±7.7 cm, peak height velocity (PHV) as 10.1±1.6 cm/year, total height increment at puberty as 26.4±4.3 cm, and duration of puberty as 4.9±0.6 yrs. Significant relationships were found between height at onset of puberty and final height and between duration of puberty and final height, while BMI at onset and age of onset of puberty and duration of puberty were negatively correlated. In a second study by the same team conducted on 1020 girls of ages 8-18 yrs, 101 of whom reached their final height, age of onset of puberty was 10.1±1.0 yrs, height at onset 141.7±7.6 cm, duration of puberty 4.9±1.2 yrs, menarche age 12.2±0.9 yrs, PHV 8.5±1.0 cm/yr and total height gained 16.0±3.9 cm. A positive correlation was found between height at onset of puberty and final height. Age of onset of puberty was negatively correlated with height and body weight at onset (129).

**Anthropometric Studies on Adults**

The Turkish Anthropometric Survey (130,131,132) initiated with Mustafa Kemal Atatürk’s orders, organized by Professor Afet İnan and designed with contributions from a number of researchers, stands out as the largest and most comprehensive anthropometric study on the adult Turkish population. Prior to this survey which was reported in 1937, a number of studies aiming to establish the racial origin of the Turkish population and the links between the Turkish and European populations had been performed on small groups living in Anatolia, in Thrace and in the Balkans. Pittard (133), in his paper entitled “Les peoples de Balkans”, gives data on stature in Ottoman Turks. He also mentions that in previous anthropological texts, mean stature in Turks was reported to be around 167-168 cm, with 63% of the population being taller than the normal mean of 165 cm suggested by Topinard in 1885 (cited in Pittard’s paper) for European men. Also citing the measurements performed by Chantre on Ottomans living in Asia Minor, who found a mean stature of 171 cm, the author states that the Ottomans living in Anatolia represent a less mixed population than those on the European side and therefore are taller. Pittard explains the variability in stature in Ottoman Turks by emphasizing the fact that the Turkish population represents a heterogeneous mixture of racial groups. He also states that a similar variability is encountered in cranial structure. The author found a mean cephalic index of 82.2 in the Ottoman population. According to this index, 49% of the study group was classified as brachycephalic, 26% as dolichocephalic, and the remainder as mesaticephalic. Pittard claims that the Asian Turks tend to be more brachycephalic compared to the European Turks and therefore it is plausible that this group originated from the brachycephalic communities in Central Asia.

Pittard et al (134), in a subsequent study, measured 210 Turkish soldiers in Anatolia, using 20 different anthropological parameters. He reported a mean stature of 171 cm in this group, a higher value than those he had reported previously. He also found that 93.3% of the individuals were taller than the accepted normal mean. Pittard also found a mean cephalic index of 85.1% (73.3% of the subjects were brachycephalic) in this group. Mean values for other parameters were: trunk length 89.3 cm; trunk/height ratio 52.0%; leg length 81.7 cm, leg/trunk ratio 91.1%; and span/height ratio 102.4%. The author also reported that the Anatolian Turks had straight or aquiline noses and 75% could be classified as leptorhinian and that they had small ears, with eyes and hair which were generally of dark color. The author goes on to state that features such as short stature, a long head, and flat nose were rare in these individuals.

Kansu (135,136), aiming to establish the differences between Asian and European Turks, compared 53 anthropometric parameters in 100 male subjects of ages 24-28 years in each group. Mean supine length was 167.9 cm and 167.3 cm in the Asian and European subjects, respectively. The author, referring to the Topinard classification which accepts 165 cm as the normal mean for male stature, concluded that the Turks were a population with a stature above the mean. Cephalic, facial and nasal indices, in respective order, were 84.2, 87.3, 65.8, in the Anatolian Turks and 82.6, 87.9 and 66.8 in the European Turks. The author also reported more brachycephalic individuals and relatively shorter leg lengths in the Anatolian Turk group. Kansu is also the author of “A Guide for Anthropological Investigations” (137) in which he gives a detailed description of instruments used in physical anthropology and the measurements used in man.

Inan (138) reported measurements on 200 Turkish women living in a district near Ankara. Mean values were 155 cm for stature, 82.6 cm for sitting height, and 78.9 cm for lower limb length. Of these subjects, 20.5% were within the normal range for stature (155 to 159.9 cm), 30% were tall (>160 cm), and 49.5% were below the stated normal range (<154.9 cm). By skelic index, the women subjects were defined as mesatiskelic and brachy-hypsicephalic. The nose was concave in shape in 62%, the eyes were of intermediary color in 63%, and the hair was intermediary in color in 56%. Mongolic eye contour was not observed in any subject. This author also gives a review of early anthropometric studies on the Turkish population, mentions that these were restricted to male subjects and refers to the works of Weissbach (44 subjects), Bossanovitch (42 subjects in Bulgaria), Eliseff (288 subjects in Asia minor), Chantre (288 subject in Asia minor), Von Luschan (40 subjects of the Bektaşi religious order), and Hauschild and Wangenseil (272 Anatolian Turks). Mean statures reported by these authors were 162.2 cm, 166.2 cm, 167 cm, 171 cm, 166 cm, and 167.2 cm, respectively. Inan also refers to the works of Pittard and of Kansu.

The Turkish Anthropometric Survey (130,131,132) can be cited as an important contribution to anthropological sources on the Turkish population. The study was carried out on adult subjects living in cities, small towns, and villages in ten regions in Anatolia and in Thrace. The data were collected over a period of four months by a team of twenty-three persons consisting of doctors, male nurses, and teachers of physical education. The team was exposed to one full week of training by Kansu. The tools for the anthropometric measurements were supplied by a Swiss firm and the design of the classification tables was done by Pittard. The physical characteristics of the study group which consisted of 39465 males and 20263 females can be summarized as follows: mean stature was 165.2 cm in Turkish men and 152.2 in the women. In both sexes, stature was relatively greater in the Eastern Anatolian regions. Fifty-two percent (49.6% in the Western and 53.6% in the Eastern regions) of Turkish men were taller than 165 cm, which, by Topinard’s definition (1885), was considered the mean for normal stature in males. On the other hand, 55.7% (57.9 in the western and 51.8% in the eastern regions) of the women were below the normal mean, when this same author’s figure of 153 cm was taken as the normal mean stature for women. Mean values for skelic index were 93.9 for the men and 83.3 for the women, thus the men were classified as macroskelic, while the women were mesatiskelic. Mean values for cephalic index were 83.3% in the men and 83.8% in the women. The proportion of brachycephalics was 63.9% in the men and 65.5% in the women according to Deniker’s classification. By Topinard’s criteria, these proportions were 75.6% and 77.7%. Mean values for nasal index were 65.0 for the men and 64.0 for the women, the proportion of leptorhinians being 71.2% and 76.7%, in respective order. The nose shape was defined as straight in the majority of the subjects. The frequency of a mongoloid slant in the eyes was 5%. Skin and eye colors varied between medium and fair. Hair was generally of chestnut color.

Gungor in 1939 (139) published the results of his anthropometric study on 40 male and 40 female adult Yörük (pastoral nomadic Turks) subjects living in the proximity of Denizli (a town in the Aegean region). These subjects were taller than those in Kansu’s and Inan’s series (135,136,138). Mean stature of the men was 170.0 cm and that of the women was 158.5 cm. The subjects were also described as leptorhinian and hypsicephalic. By cephalic index, most men were classified as dolichocephalic and the women as mesaticephalic. By skelic index, the women were generally mesatiskelic, while the men were macroskelic. By facial index, hyperleptoprosopy was the most frequent typology, leptoprosopy occupying second rank. Eye and hair color was dark or brown in these subjects.

Hertzberg (140) measured 915 Turkish, 1084 Greek and 1357 Italian soldiers in an effort to establish standards for the design of such items as oxygen masks, helmets, clothing and seats. He took 150 measurements on each individual and compared the three groups. Mean values for stature, weight, and sitting height were 169.3 cm, 64.6 kg, and 89.7 cm in the Turkish, 170.5 cm, 67.0 kg, and 90.2 cm in the Greek, and 170.6 cm, 70.3 kg, and 90.2 cm in the Italian subjects, in respective order.

In later years, Saatcioglu (141,142), analyzing the results of Inan’s survey data, showed a positive correlation in both sexes between stature and both head length and head width. This investigator also reported a study on 568 male and 556 female subjects, assessing stature, chest circumference and cephalic index in these subjects who were classified as university students, skilled workers and unskilled workers. The author reported significant variations in stature by socioeconomic level.

Emekli (143,144), in a study performed in 1966 on a randomized sample of 1865 adult young men from various regions in Turkey and of different occupational groups, reported a mean stature of 166.6 cm and a positive secular trend of 14 mm from 1937 values (130,131,132). Mean skelic index was 87.6 in this group and in contrast to the 1937 study results, typology in the majority of the subjects was mesatiskelic and tended to be brachyskelic in some. Mean cephalic index was 83.9, the proportion of brachycephalics being 70.6%. In this study, similar to previous publications, frequency of tall stature and dolichocephaly was greater in subjects from the Eastern and Southeastern regions of Anatoli. He also reported stature, trunk length, body weight and chest circumference measurements and muscle strength measured by dynamometry in 525 army recruits. In another study (145), the same author reported a significant positive correlation between stature and foot length.

A number of studies, aiming to establish anthropometric norms in the Turkish adult population were published in the 1980s (146,147,148,149,150,151,152,153,154,155,156,157, 158). Kuran and Sahmay (146) performed 15 anthropometric measurements on 200 women and reported higher head/stature and hand length/stature and lower foot length/stature and shoulder width/stature ratios in Turkish women as compared to European and North American women. A head length/stature ratio of 1/7.08 in adult women was reported by Sahmay (147). Muftuoglu and Gurun also measured some body parts and stature in adult male subjects (148). Gurun and Kuran (149) reported a mean value of 1/7.6 for head/stature ratio in both sexes. Muftuoglu et al (150) measured facial dimensions in 100 adults and 100 newborns and reported a ratio of 1/13.84 for face width/face length in adult males. This ratio was 1/12.11 in adult females, 1/10.05 in male newborns, and 1/9.68 in female newborns. Facial type was reported as mesoprosopic in 85.1% of adult males, and 84.4% of adult women were classified as europrosopic. Arı et al (151, 152) reported that leptoprosopy (ratio between 0.90 and 0.95) was the dominant feature in 232 girls and 336 males between 17 and 25 years of age. Cireli et al (153) reported anthropometric measurements in university students. Cireli et al (154) also measured auricular length in 624 male and 376 female students aged between 18 and 23 years and reported that the ears were of small size in the females and of medium size in the males. Foot measurements revealed that Turkish men and women have shorter and wider feet than many European groups (156,157). Vural (158) measured pelvic dimensions in Turkish women and reported higher values compared to European women.

Twenty different anthropometric measurements were performed on 400 medical students in Istanbul (159, 160). In the women, mean and SD values for stature, sitting height and body weight were 160.2±5.7 cm, 86.6±8.4 cm and 56.6±5.7 kg, respectively. These values were 174.0±1.6 cm, 94.4±34.2 cm and 69.5±9.1 kg in men, in respective order. The majority of the students were brachycephalic, had high trunk/stature ratios and narrow faces. In two other studies on university students, a mean stature of 164.1 cm and 163.8 cm in the women and of 175.9 and 176.4 cm in the men was reported (148,149). These relatively recent studies show an overall increase in stature of approximately 10 cm since the 1937 report. However, this statement can be valid only by comparing groups matched for socioeconomic background. For this, there is a need to perform measurements on a population representing all socioeconomic groups.

In a recent study, Ozer et al (161) report measurements on human skeletons dating as far back as the Neolithic and Chalcolithic periods in the history of man and compare them to more recent measurements. Based on these measurements, the authors report periods of positive and negative secular trends in man’s history. Some other studies have aimed to evaluate the possibility of estimating stature from measurements of other body parts. Of these, studies aiming to assess stature from measurements of knee length are of clinical importance for geriatric and/or incapacitated patients (162,163,164). Some other studies have been conducted to be of guidance to orthopedists, ophthalmologists, radiologists in their clinical practice (165,166,167,168,169,170,171,172). Some studies in Turkish adults have been designed to assess the methods of assessment of obesity (173,174,175,176). Studies on prevalence of obesity have also been reported (177). One such study was conducted in the city of Sivas on 500 men and 500 women of ages 20 to 65 years and shows that women are more prone to be obese. In this study, mean height was 168.0 in the men and 154.3 cm in the women. Mean weight was 78.9 kg in the men and 72.1 kg in the women. Mean values for BMI were 28.0 in men and 30.4 in women. Skinfold thickness values and WC/hip circumference (HC) ratios were also higher in the women (178). Another study covering 26 499 individuals (63% women),conducted jointly by a team from several universities, the State Institute of Statistics and the Ministry of Health and repeated after an interval of 12 years showed that, along with significant increases in frequency of overt diabetes and impaired glucose tolerance, body weight had increased by 8 kg in men and 6 kg in women, WC by 7 cm in men and 5 cm in women, and HC by 2 cm in men and 1 cm in women. Height had increased by 1 cm in both sexes (179,180).

**Comments**

This historical review of the available anthropometric data on the Turkish population provides some insights into the growth status of the children of this region over the past fifty years as well as on various anthropometric parameters pertaining to the adults.

In the early studies, the measurements on children aspired to establish the status quo of level of growth at different ages in the two sexes. In the 1960s, the impact of the environment on growth began to gain much attention, as seen by many studies documenting differences in growth attainment by socioeconomic level. The quality of the data in some of these studies fails to meet the standards required in scientific publications today. Information on methodology relating to sample selection, age grouping, and measurement technique is not given. The results, presented only as mean values, fail to provide information on within group variation and do not allow statistical comparisons. The majority of the studies are limited to children living in cities. Despite these limitations, the data still indicate that a significant positive secular trend has occurred over the past fifty years in the growth of Turkish children, reflecting the improvement in the standard of living which has occurred in this country in the past decades (181). It is also of interest that the secular trend is not noticeable to a significant degree in children of the upper echelons of society. For this reason, the growth charts based on the measurements of high bracket children born four decades ago appear to practically retain their validity for today’s children.

The early studies on adults were directed mainly to document differences among ethnic groups. We believe that in the first half of 20th century, the anthropologists involved in these studies were influenced by concepts prevailing among western scholars of the 19th century, which, following a hereditary viewpoint, concentrated mainly on racial differences. Thus, the impact of polymorphic variation is hardly mentioned and the emphasis is on polytypic variation (5,182,183). Also, in these early studies, there is no mention of the significant secular trend in height which occurred in Europe in the first 30 years of this century (4,184). It is difficult to justify the use of Topinard’s figures as reference points to classify the subjects by stature in the studies performed in the late thirties. In many of these publications, Turks living in Eastern Anatolia are reported as being taller than those in the western European parts of the country. Whether this biological difference can be attributed to “less mixing”, without mention of other factors, is debatable. Particularly in cranial measurements and the indices derived from these measurements, there is no mention in the early studies of the relation of head size and shape to body size, nor of nutritional and other environmental factors influencing both head and body size. As early as 1899, Boas showed that the cranial index was not a dependable standard in classifying individuals, since this index varied widely both among adults of a single group and within the life of an individual (2). He also wrote that head shape could change in a single generation of altered environment. Today, it is well known that undernutrition interferes with cell proliferation throughout the body including the brain and that organ size as well as stature are affected by environmental factors, as shown by smaller head size in low socioeconomic groups (185). Despite these critical remarks, the 1937 Turkish Anthropometric Survey in particular retains its value as an important document which can be used as a baseline to assess changes in body size and shape that must have occurred over time. Considering the paucity of published work on measurements on representative groups of adults in recent years, there is a need to define with greater precision the anthropometric characteristics of the present adult population in Turkey.

**Acknowledgements**

The authors acknowledge the support of Professor Nuran Yıldırım, a staff member of the History of Medicine and Ethics Department of the Istanbul Faculty of Medicine and that of Professor Zuhal Özaydın, a staff member of the History of Medicine and Ethics Department of the Cerrahpaşa Faculty of Medicine, in finding and interpreting the Ottoman sources cited in this paper. in this paper.
